# Primary lymphoma of bone in children: Three case reports and literature review

**DOI:** 10.1002/pdi3.15

**Published:** 2023-06-10

**Authors:** Man Zhang, Jianwen Xiao, Xing Liu, Ziyuan Zhang, Xiang Li, MingYan Shi, Peikang Wang, Xinkai Zhang, Hailun Yao

**Affiliations:** ^1^ Children's Hospital of Chongqing Medical University Chongqing China

**Keywords:** children, gene mutation, imaging finding, pathology finding, primary lymphoma of bone

## Abstract

Primary lymphoma of the bone (PLB) is a rare form of extranodal lymphoma. The clinical features and imaging findings of PLB are not specific. Histological examination and immunophenotyping is the gold standard for diagnosis. We reported three cases, all diagnosed with B‐cell lymphoblastic lymphoma. The first patient is a 4‐year‐old boy with 6 months history of PLB. Lytic permeative areas with soft‐tissue masses showed on imaging. A mutation of the FANCA gene of this patient was detected through whole exome sequencing (WES). The second patient is a 17‐year‐old boy with multiple areas of pain over 2 months. Flurodeoxyglucose positron emission tomography/computed tomography showed that the density of multiple bones was slightly increased. WES revealed a heterozygous splice site variation in the SBDS gene. The third patient is a 3‐year‐old boy with swelling and pain of the left knee joint and fever for 43 days. An oval like lesion area appeared on imaging. The display of case details helps to diagnose and understand PLB. The correlation between the occurrence and progression of the disease and FANCA gene and SBDS gene mutations remains to be studied.

## 1 INTRODUCTION

Primary lymphoma of the bone (PLB) is rare^1^; besides, the definition of it has no consensus at present.^2^ Followed by occasional pathology fracture, bony pain and tumor masses are the most common features in patients. The imaging findings of primary bone lymphoma are not specific.^3^ Positron emission tomography/computed tomography (PET/CT) has its advantages on both pretreatment staging and evaluating response to treatment. The diagnosis of PLB needs to combine clinical manifestation, imaging data, and biopsy for histological examination and immunophenotyping, which is the gold standard of PLB diagnosis. In this paper, we report three child patients diagnosed with PLB.

## 2 CASE PRESENTATION

The recruitment and use of clinical data were approved by the Institute Ethics Committee. Informed consent was obtained from the patients for publication of this case report.

### 2.1 Clinical features

We noticed that some patients diagnosed with lymphoma presented with bony pain. We selected three cases that involved the bone as the primary site without visceral or lymph node involvement among patients with lymphoma in the Children's Hospital of Chongqing Medical University between 2017 and 2022. The patients' age range varied from 3 to 17 and all patients were male. They mainly suffered from unexplained joint pain with or without fever and were referred to our hospital for diagnosis and treatment. In two patients (case 1 and case 3), imaging test revealed an abnormal lesion area on the femur or hip bone. They have no recurrence so far after they received chemotherapy with the modified BFM‐95 regimen. A summary of clinical details of the three patients is revealed in Table S1.

### 2.2 Imaging findings

**FIGURE 1:**
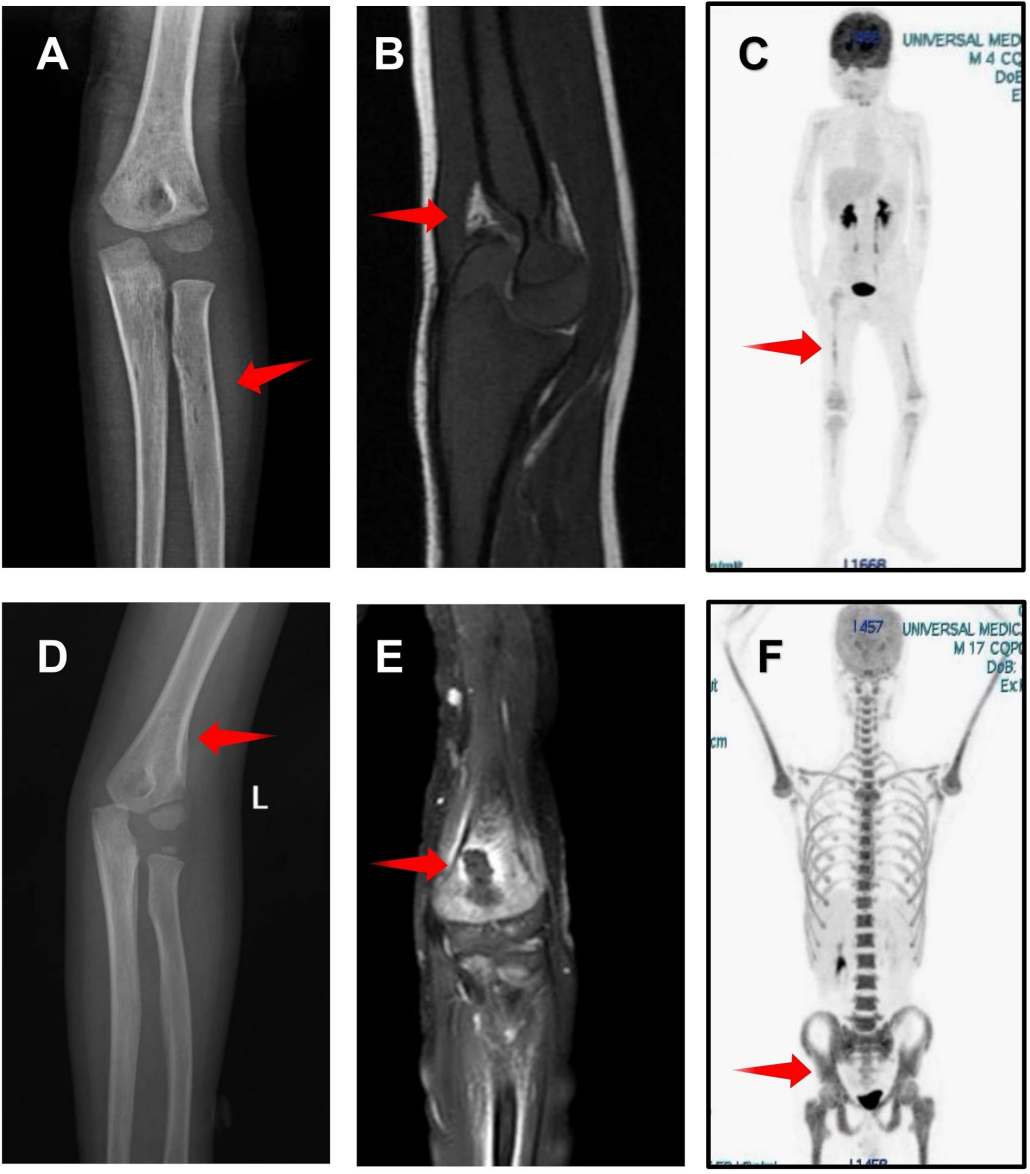
Imaging data of patients. (A) Anteroposterior radiograph of the left elbow joint of case 1. (B) T1 sagittal of the left elbow joint of case 1. (C) PET/CT of case 1 after the first session of chemotherapy. (D) Anteroposterior radiograph of the left elbow joint of case 3. (E) T1 sagittal of the left elbow joint of case 3. (F) PET/CT of case 2. PET/CT, positron emission tomography/computed tomography.

Obvious osteopathic changes can be seen in two cases (cases 1 and 3). Radiograph of case 1 reveals lytic permeative, worm‐eaten areas. Soft‐tissue masses around the distal left humerus is shown on MRI. The PET/CT of case 1 shows the density of multiple bone (or bone marrow), including bilateral long bones of lower limbs, bilateral humerus, and left ulna in the whole body, which was slightly increased after the first session of chemotherapy. A little periosteal reaction was seen on the distal end of the left humerus through a radiograph of case 3 and an oval like lesion area appeared at the same site through MRI. The other case (case 2) showed quantity hypermetabolic lesions on the occipital bone, mandible, clavicle, scapula, ribs, sternum, spine, and pelvis through PET‐CT, while CT did not find obvious lesion areas in the PET/CT‐indicted location. The imaging features of the three patients are shown in Figure 1.

### 2.3 Pathology findings

**FIGURE 2:**
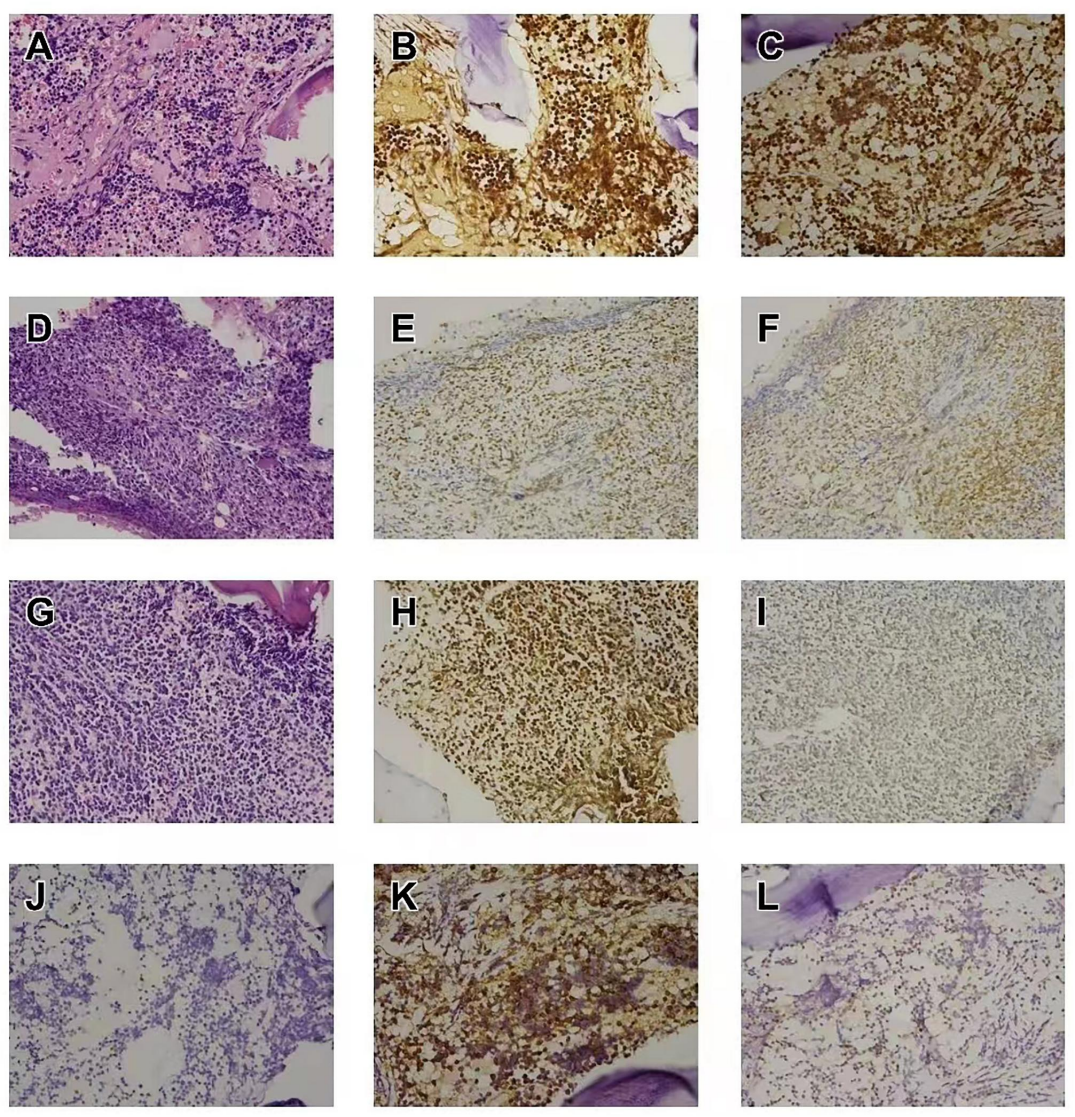
(A) Hematoxylin and eosin staining of case 1. (B, C) Immunohistochemistry microphotograph of case 1 shows that tumor cells are positive for PAX‐5 and TdT. (D) Hematoxylin and eosin staining of case 2. (E, F) Immunohistochemistry microphotograph of case 2 shows that tumor cells are positive for PAX‐5 and TdT. (G) Hematoxylin and eosin staining of case 3. (H, I) Immunohistochemistry microphotograph of case 3 shows that tumor cells are positive for PAX‐5 and negative for TdT. (J–L) Immunohistochemistry microphotograph of case 1 shows that tumor cells are positive for CD7, CD34, and CD99.

Histologically, bone marrow biopsy of all the cases shows focal or scattered distribution of naive lymphocytes. Immunophenotyping shows positivity for PAX‐5, TdT, CD7, CD34, and CD99 in case 1. Bone marrow biopsy of case 2 shows naive lymphocytes proliferated actively and collagen fiber hyperplasia can be seen in bone marrow stroma. Moreover, immunophenotyping shows positivity for PAX‐5 and TdT. Histological analysis of case 3 shows a few small round cells with sparse cytoplasm and hyperchromatic nuclei, and immunophenotyping shows positivity for PAX‐5, while negativity for TdT. The pathology features of three patients are shown in Figure 2.

### 2.4 WES results

**FIGURE 3:**
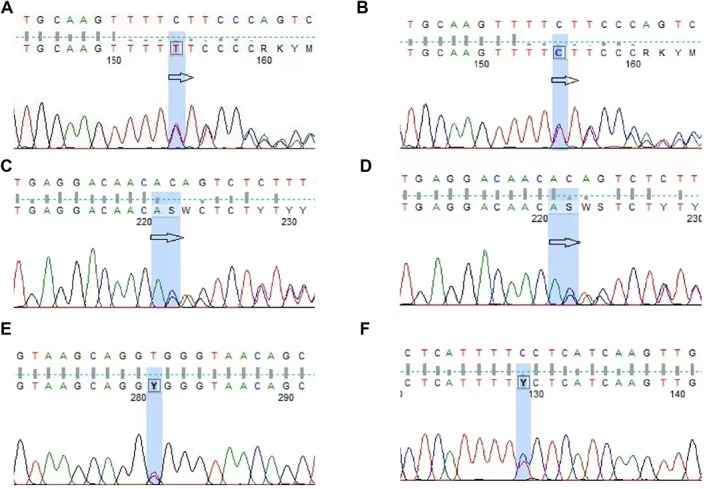
(A) The WES result of case 1 showed the FANCA gene mutation. (B) The WES result showed that the mother of case 1 had the FANCA gene mutation. (C) The WES result of case 1 showed the CD36 gene mutation. (D) The WES result showed that the mother of case 1 had the CD36 gene mutation. (E) The WES result of case 2 showed the SBDS gene mutation. (F) The WES result of case 2 showed the G6PC gene mutation. WES, whole exome sequencing.

To investigate the genetic bases of PLB, whole exome sequencing (WES) was performed for the two cases with bone marrow samples (except case 3 due to the lack of biological parents' information). The WES results showed that case 1 and his mother all had the FANCA c.2546del heterozygous mutation and CD36 c.332_333del heterozygous mutation (Figure 3A–D). Case 2 had SBDS c.258+2T>C heterozygous mutation and G6PC c.532C>T heterozygous mutation (Figure 3E–F).

## 3 DISCUSSION

Primary lymphoma of the bone (PLB) is a rare form of extra‐nodal lymphoma, constituting approximately 5% of extranodal lymphomas, <1% of all nonHodgkin lymphomas (NHLs) and 3%–7% of all malignant bone tumors.^1^ In children, PLB accounts for a higher proportion of NHLs, comprising 2%–9% of the cases.^4^ Glotzbecker et al. identified childhood PLB to represent 3.3% of NHL cases, with a male to female ratio of 1.65:1 and a mean age of 11.3 years in a series of 15 patients and review of 91 additional cases from the literature.^5^ The definition and classification of PLB have changed many times in the past several years. According to the 2020 World Health Organization, classification of tumors of the bone and primary NHL of the bone was renamed as malignant lymphoma, nonHodgkin. There is no change in definition: (1) a single skeletal tumor without regional lymph node involvement or (2) multiple bone lesions without visceral or lymph node involvement. However, there is no categorization of cases with multifocal osseous disease or cases with concomitant soft tissue, visceral, and/or lymph nodal infiltration and that is why there is no concurrence in the definition of PLB. The etiology of PLB is unclear.

Bony pain is the most common presenting symptom (80%–95%) of PLB, tumor mass is present in 30%–40% of the cases and pathological fracture in 15%–20%.^6^ All patients reported in our case had multisite bony pain. The first patient presented with bilateral elbow joints and knee joints pain. The second patient had left shoulder and right costal arch pain, and the third patient had left knee joint and left elbow joint pain.

Radiographic features of PLB are various. Radiological appearance can be normal, lytic, mixed lytic/sclerotic, or sclerotic.^3^ One such pattern described as being typical and most common of PLB is a solitary lytic lesion near the end of a long bone that has a permeative or moth‐eaten pattern of destruction and aggressive periosteal reaction.^7^ It is worth noting that this typical pattern is not specific and differential diagnosis is varied.

The radiographic features of three patients are diverse from each other. On radiographs of the first patient, a lytic permeative area within the distal left humerus surrounded with soft tissue masses without extensive cortical destruction revealed on MR imaging was observed. The PET/CT of the second patient showed that the density of multiple bone (or bone marrow) in the whole body was slightly increased. The radiographs of the third patient revealed the periosteal reaction surrounded with swollen soft tissue as seen on MR imaging.

Not only imaging examinations but also WES were done on our patients. Here are two significant findings: A heterozygous frameshift mutation in the FANCA gene of the first patient and a heterozygous splice site variation in the SBDS gene of the second patient were detected.

FANCA gene on chromosome 16q24 is one of the associated genes of Fanconi anemia (FA). Soulier et al.^8^ noted that the FANCA, ‐C, ‐E, ‐F, ‐G, and ‐L proteins are part of a nuclear multiprotein core complex, which triggers activating monoubiquitination of the FANCD2 protein during the S phase of the growth cycle and after exposure to DNA crosslinking agents. The FA/BRCA pathway is involved in the repair of DNA damage.

SBDS gene on chromosome 7q11 is widely expressed in many cells and groups such as the pancreas and bone marrow and so on and encodes a highly conserved protein containing 250 amino acids. This highly conserved protein is a multifunctional protein affecting bone marrow hematopoietic microenvironment, mesenchymal stem cell function,^9^ and other biological processes. Whether these two gene mutations can cause PLB and how to cause PLB remain to be studied.

Owing to the fact that clinical manifestations and imaging are nonspecific, the gold standard for diagnosis of PLB is biopsy for histological examination and immunophenotyping. The most common pathological type is primary bone diffuse large B‐cell lymphomas (PBDLBCL) with a rare occurrence of follicular, marginal zone, anaplastic large cell, Hodgkin, and T ‐cell lymphomas.^4^, ^6^ The tumor cells in PBDLBCL are positive for CD45, CD20, CD79a, and PAX5 (B‐cell markers).^10^ Other subtypes of PLB are not described here. The first and the second patients were diagnosed with primary B‐cell lymphoblastic lymphoma of the bone. The third patient was diagnosed with B‐cell lymphoma.

Several studies have suggested that a combination of chemotherapy and radiotherapy was the best treatment for patients with PLB. Beal et al.^11^ reported that progression free survival and overall survival of PLB subtype with CD20‐positive B‐cell lymphoma have been greatly improved by combining rituximab. Rituximab seems to have a positive effect but more experiments are needed to prove it.

With PLB cases reported in children being sparse, the aims of this article are to increase the awareness of the disease and provide a differential diagnosis of bony pain, which is a common symptom in children. Besides, we raised a question whether the gene mutations of FANCA gene and SBDS gene are associated with PLB.

## AUTHOR CONTRIBUTIONS

**Man Zhang**: Data curation, Writing—review & editing, Writing—original draft, Conceptualization, Methodology. **Jianwen Xiao**: Resources, Methodology, Conceptualization, Data curation. **Xing Liu**: Conceptualization, Methodology, Resources. **Ziyuan Zhang**: Writing—original draft, Formal analysis. **Xiang Li**: Visualization, Validation. **Mingyan Shi**: Visualization, Validation. **Peikang Wang**: Investigation. **Xinkai Zhang**: Validation. **Hailun Yao**: Investigation.

## CONFLICT OF INTEREST STATEMENT

This research did not receive any specific grant from funding agencies in the public, commercial, or not‐for‐profit sectors. All authors declare no conflict of interests.

## ETHICS STATEMENT

The recruitment and use of clinical data were approved by the Institute Ethics Committee. Informed consent was obtained from the patients for publication of this case report.

## Supporting information

Table S1

## Data Availability

Data sharing not applicable to this article as no datasets were generated or analyzed during the current study.
