# Supplementary material for: Primary lymphoma of bone in children: Three case reports and literature review
Source: Pediatr Discov. 2023 Jun 10;1(1):e15. doi: 10.1002/pdi3.15 (PMC12118206; doi:10.1002/pdi3.15)
Supplement: Supplementary file 1 — Table S1 [file PDI3-1-e15-s001.docx]

| Case no. | Age/  Gender | Symptom | Imaging | Histopathologic  features | Immunohistochemistry | Treatment | Recurrence |
| --- | --- | --- | --- | --- | --- | --- | --- |
| 1 | 4/M | Left elbow joint pain with fever for 6 months | plain radiography: lytic permeative areas on the distal left humerus.  MRI: soft-tissue masses on left humerus | Naïve cells showed a scattered or focal distribution, small, round or oval. | Positive for PAX-5, CD5, TdT, CD34, CD99, CD7.  Negative for MPO, syn, myogenin,NKX2.2. | Modified BFM-LBL-95 Regimen | No |
| 2 | 17/M | left shoulder and right costal arch pain with intermittent fever over 2 months | 18F-FDG PET/CT/MR：Quantity hyper-metabolic lesions on occipital bone, mandible, clavicle, scapula, ribs, sternum, spine, pelvis. | Bone marrow  biopsy showed naïve lymphocytes proliferated actively and collagen fiber hyperplasia can be seen in bone marrow stroma. | Positive for CD34, CD61, CD3, CD10, CD79a, TdT.  Negative for CD117. | Modified BFM-95 Regimen | No |
| 3 | 3/M | developed swelling and pain of his left knee joint with elevated skin temperature, walking claudication and fever 43 days | plain radiography: a little periosteal reaction seen on the distal end of the left humerus and the proximal end of the bilateral ulna.  MRI: an oval like lesion area appeared at the distal end of the left humerus, dark on T1-weighted images and bright on T2-weighted images. | A few small round cells with sparse cytoplasm and hyperchromatic nuclei can be seen. | Positive for PAX-5, Vin.  Negative for CD7, CD99, TdT. | Modified BFM-95 Regimen | No |

Table 1 Clinical , imaging , and pathology features

M male, MRI magnetic resonance imaging, PET positron emission tomography scan, FGD fluorodeoxyglucose, PAX-5 paired box protein 5, TdT terminal deoxynucleotidyltransferase, BFM-95 Regimen NHL-Berlin-Frankfurt-Münster-95 Regimen.
